# AEG-1 Promotes Anoikis Resistance and Orientation Chemotaxis in Hepatocellular Carcinoma Cells

**DOI:** 10.1371/journal.pone.0100372

**Published:** 2014-06-18

**Authors:** Zhenzhen Zhou, Huan Deng, Wei Yan, Min Luo, Wei Tu, Yujia Xia, Jiayi He, Ping Han, Yu Fu, De'an Tian

**Affiliations:** 1 Department of Gastroenterology, Tongji Hospital, Tongji Medical College, Huazhong University of Science and Technology, Wuhan, China; 2 Department of Gastroenterology, Union Hospital, Tongji Medical College, Huazhong University of Science and Technology, Wuhan, China; University of Quebec at Trois-Rivieres, Canada

## Abstract

Metastasis contributes to the poor prognosis of hepatocellular carcinoma (HCC). Anoikis resistance and orientation chemotaxis are two important and sequential events in tumor cell metastasis. The process of tumor metastasis is known to be regulated by AEG-1, an important oncogene that plays a critical role in tumor metastasis, though the effects of this oncogene on anoikis resistance and orientation chemotaxis in HCC cells are currently unknown. To directly assess the role of AEG-1 in these processes, we up-regulated AEG-1 expression via exogenous transfection in SMMC-7721 cells, which express low endogenous levels of AEG-1; and down-regulated AEG-1 expression via siRNA-mediated knockdown in MHCC-97H and HCC-LM3 cells, which express high endogenous levels of AEG-1. Our data directly demonstrate that AEG-1 promotes cell growth as assessed by cell proliferation/viability and cell cycle analysis. Furthermore, the prevention of anoikis by AEG-1 correlates with decreased activation of caspase-3. AEG-1-dependent anoikis resistance is activated via the PI3K/Akt pathway and is characterized by the regulation of Bcl-2 and Bad. The PI3K inhibitor LY294002 reverses the AEG-1 dependent effects on Akt phosphorylation, Bcl-2 expression and anoikis resistance. AEG-1 also promotes orientation chemotaxis of suspension-cultured cells towards supernatant from Human Pulmonary Microvascular Endothelial Cells (HPMECs). Our results show that AEG-1 activates the expression of the metastasis-associated chemokine receptor CXCR4, and that its ligand, CXCL12, is secreted by HPMECs. Furthermore, the CXCR4 antoagonist AMD3100 decreases AEG-1-induced orientation chemotaxis. These results define a pathway by which AEG-1 regulates anoikis resistance and orientation chemotaxis during HCC cell metastasis.

## Introduction

Hepatocellular carcinoma (HCC) is one of the 5 most common cancers and the third leading cause of cancer-related death worldwide [1]. Metastasis, rather than the primary tumor per se, contributes to the poor prognosis of HCC [2]. Tumor metastasis is a multi-step biological process in which tumor cells invade the extracellular matrix and cell layers, intravasate into vessels, survive and migrate to targeting organs, arrest at distant organs, extravasate into the parenchyma of tissues, adapt to these foreign microenvironments, and finally form micrometastases [3]. Metastasis represents a highly organized, non-random and organ-selective process [4]. A notable feature of this process is the variation in metastatic organ tropism displayed by different types of cancer [5]–[7]. For example, over 60% of advanced stage breast cancer patients suffer from bone metastasis, but they rarely develop kidney, stomach, spleen or uterine metastases [8], [9]. Furthermore, 55% of advanced stage HCC patients suffer from lung metastasis, but metastases to other distant organs, such as the brain and bone, are relatively rare [10].

An important step in the process of metastasis occurs when tumor cells reside in the lumina of vessels: anoikis occurs for the majority of cells due to the disruption of epithelial tumor cell-extracellular matrix interactions [11]; however, the circulating tumor cells that acquire the ability to survive in the absence of extracellular matrix interactions migrate toward specific organs with the help of selected chemokines [12]. Therefore, the processes of anoikis resistance and orientation chemotaxis play key roles in the metastasis of cancer cells. However, few studies have focused on the roles of anoikis resistance and orientation chemotaxis in HCC metastasis.

Astrocyte elevated gene-1 (AEG-1, also named metadherin [MTDH] or Lyric) has been established as an oncogene in a variety of cancers [13]–[17]. AEG-1 was first cloned as an HIV and TNF-α-inducible gene in primary human fetal astrocytes (PHFA) [18]; however, recently, AEG-1 has been shown to play a vital role in tumor progression. AEG-1 synergizes with the oncogenic Ha-ras to enhance soft agar colony formation of non-tumorigenic immortalized melanocytes [19]. AEG-1 inhibits serum starvation-induced apoptosis by activating PI3K/Akt signaling in PHFA cells [20]. Knockdown of AEG-1 inhibits prostate cancer progression though the downregulation of Akt activity and upregulation of forkhead box (FOXO) 3a activity [15]. In addition, AEG-1 mediates lung metastasis of human breast cancer by enhancing the adhesion of tumor cells to lung microvascular endothelial cells and promotes chemoresistance [21]. A lung homing domain (LHD, amino acids 378–440 in mouse or 381–443 in human) was identified in AEG-1 that can mediate lung metastasis of breast cancer [13]. Furthermore, we previously documented that the expression of AEG-1 in HCC cell lines is positively related to orientation chemotaxis towards human pulmonary microvascular endothelial cells (HPMECs) [22]. However, a direct demonstration of the role of AEG-1 in anoikis resistance and orientation chemotaxis has not been characterized in HCC cells.

Recent studies indicate that both the death receptor-mediated extrinsic pathway and the mitochondrial-mediated intrinsic pathway contribute to anoikis [23], [24]. Activation of PI3K/Akt and MAPK signaling pathways enable cells to develop resistance to anoikis [25], [26]. The oncogene TrkB and the tumor suppressor gene PTEN also participate in the regulation of anoikis [27], [28]. Additionally, the process of anoikis is regulated by proteins of the Bcl-2 family, including anti-apoptotic proteins Bcl-2, Bcl-xL, and Mcl-1; and pro-apoptotic BH3-only proteins Bid, Bad, and Bim, as well as Bax, Bak, and Bok [29]. Moreover, accumulating studies indicate that epithelial-mesenchymal transition (EMT), autophagy and ROS are associated with anoikis [30]–[32].

Another additional focus of metastasis research involves the CXCR chemokine receptor family. CXCR4 is seven-transmembrane, G protein-coupled receptor that is found to be expressed in many human cancer cells. High expression of the ligand for CXCR4, CXCL12/SDF-1, is detected in organs that are usually the primary destinations of metastasis for these cancers [33]–[35]. In addition to their roles in chemotaxis, CXCR4 and CXCL12 have been shown to regulate anoikis in breast and colorectal cancers [36], [37]. However, the effect of AEG-1 on CXCR4 has not yet been determined.

In the present study, we explored the role of AEG-1 in HCC cells in the resistance to anoikis and orientation chemotaxis toward HPMECs. We demonstrate that anoikis occurs, in part, through the activation of the PI3K/Akt pathway and that orientation chemotaxis occurs though the CXCR4/CXCL12 axis. These results provide a novel mechanism supporting HCC metastasis.

## Materials and Methods

### Cell lines and cell culture

Human liver cancer cell lines SMMC-7721, HepG2, MHCC-97H, and HCC-LM3 (Institute of liver diseases, Tongji Hospital, Tongji Medical College, Huazhong University of Science and Technology, Wuhan, Hubei, China) were cultured in DMEM medium (Hyclone Laboratories, Logan, UT, USA) supplemented with 10% fetal bovine serum (Gibco, Rockville, MD, USA) and were incubated in a 5% CO_2_ incubator at 37°C. Microvascular endothelial cell lines (HPMECs/human pulmonary microvascular endothelial cells, HHSECs/human hepatic sinusoidal endothelial cells, and HUVECs/human umbilical vein endothelial cells) were purchased from ScienCell biotechnology company (San Diego, CA, USA) and were cultured in Endothelial Cell Medium (ScienCell) supplemented with 10% heat-inactivated fetal bovine serum (ScienCell). Cultures were maintained at 37°C under a mixture of 95% air and 5% CO_2_.

### AEG-1 over-expression and siRNA-mediated knockdown

AEG-1 cDNA (NM_178812) was cloned into the eukaryotic expression vector pcDNA3.1(−) to up-regulate AEG-1 expression in SMMC-7721 cells. As a control, pcDNA3.1(−) was used without any cDNA insert. pcDNA3.1(−)-AEG-1 and pcDNA3.1(−) plasmids were transfected into 50%-confluent SMMC-7721 cells with Lipofectamine2000 reagent (Invitrogen, CA, USA). After 6 h of transfection, cells were washed and allowed to recover in DMEM medium supplemented with 10% fetal bovine serum. After 24 h, transfected cells were trypsinized and replated into six-well plates (1∶10) and then selected for 14 days with 600 µg/ml G418 to produce stable AEG-1-over-expressing cell lines. Five single colonies (AEG-1-5, AEG-1-8, AEG-1-11, AEG-1-12 and AEG-1-16) of stable cells were picked for further culture, and the concentration of G418 was subsequently reduced by half and maintained in continued cultivation. The establishment of AEG-1-over-expressing cells was verified by assessment of AEG-1 expression. In order to inhibit AEG-1 expression in MHCC-97H and HCC-LM3 cells, the following siRNA sequences were targeted: AEG-1-siRNA-1: 5′-GGCAGGTATCTTTGTAACTA-3′, AEG-1-siRNA-2: 5′-GCTGACTGATTCTGGTTCAT-3′. The expression levels of AEG-1 were examined 72 h after transfection.

### MTT, flow cytometry analysis, and anoikis assay

A total of 5×10^3^ cells per well were plated into a 96-well plate. Cells were cultured for 24 h, 48 h and 72 h after transfection, and cell growth was assessed by using the 3-(4, 5-dimethylthiazol-2-yl)-2, 5-diphenyltetrazolium bromide (MTT) assay (wavelength, 550 nm). For apoptosis analyses, cells were harvested and stained using a PE Annexin V apoptosis detection kit (BD Pharmingen, San Diego, CA, USA) according to the manufacturer's instructions. For cell cycle analyses, the cells were harvested, washed with PBS twice, and fixed with precooled 75% ethanol. Before flow cytometry analyses, the ethanol was removed and 500 µl of freshly made dye solution (0.05 mg/mL propidium iodide, 0.025 mg/mL RNase) was added. After staining for 30 min, the cells were subjected to flow cytometry analysis. Three replicates were performed. The anoikis assay was performed essentially as described by Frisch [38]. Briefly, flasks were pre-coated with 2% sterilized agar (Sigma, St Louis, MO, USA). Cells were trypsinized and plated onto pre-coated flasks. Suspension medium consisted of DMEM supplemented with 1% methocel and 10% FBS.

### Western blot analysis

For the Western blot analyses, cells were lysed in RIPA buffer (Sigma, St Louis, MO, USA). Protein concentrations were determined with a BCA Protein Assay Kit (Pierce, Rockford, IL, USA). Equal amounts of total proteins were separated by 10% SDS-PAGE and transferred to polyvinylidene difuoride membranes (Millipore, Bedford, MA, USA). The membranes were subsequently immunoblotted with the appropriate primary antibody. Anti-AEG-1 was purchased from Abcam Ltd. (Abcam, Cambridge, MA, USA), anti-CXCR4 was purchased from eBioscience Ltd. (eBioscience, San Diego, CA, USA), and antibodies against Akt, phosphorylated Akt (S473), caspase-3, cleaved caspase-3, Bcl-2, Bad, phosphorylated Bad (S99) and GAPDH were obtained from Epitomics company (Epitomics, CA, USA). A secondary anti-rabbit antibody (Promoter Biological Company, Wuhan, Hubei, China) was used for detection with an ECL kit (Pierce) according to the manufacturer's instructions. Bands were scanned and analyzed. Each experiment was repeated three times.

### Chemotaxis assay

The AEG-1-over-expressing and AEG-1-knockdown cells were trypsinized and plated onto flasks that were precoated with 2% agar. Suspension medium consisted of DMEM supplemented with 1% methocel and without serum. After suspension culture for 24 h, cells (5×10^4^) were loaded into the upper wells of a chamber. For CXCR4 competitive inhibition, cells were pretreated with the CXCR4 antagonist AMD3100 (Sigma) for 2 h at 37°C prior to incubation. We selected 10 µM AMD3100 as the experimental dosage because this concentration has been used in several in vitro studies investigating the role of CXCR4 in cancer [39], [40], and we also demonstrated that this concentration haves no growth inhibitory effect. The lower wells of the Boyden chamber were loaded with HPMECs supernatant containing chemoattractant. Serum-free endothelial cell medium was loaded as a control. A polyvinylpyrrolidone-free polycarbonate filter with 8 µm pores was placed on the lower wells, and then the Boyden chamber was incubated for 3 h at 37°C in 5% CO_2_. The membranes containing the invasive cells were stained with hematoxylin and mounted on slides, and the entire membrane with invading cells was counted by light microscopy. Each experiment was repeated three times.

### ELISA analysis of cytokine/chemokine

Semiconfluent human microvascular endothelial cell lines were growth arrested for 48 h in a serum-free endothelial cell medium substratum. Supernatants were then collected, centrifuged at 1500 rpm for 5 min at 4°C to remove cellular debris, and stored at −80°C until analysis was carried out by ELISA. Immunoreactive CXCL12 was quantified using ELISA kits (R&D, Emeryville, CA, USA), according to basic laboratory protocols. Each data point represents readings from a minimum of four independent assays performed in triplicate.

### Statistical Analysis

The SPSS16.0 software package was used for statistical analysis, and measurement data was presented as mean ± SD. Statistical significance of the differences was determined using a Student's t-test. *P*<0.05 was considered significant.

## Results

### AEG-1 enhances anoikis resistance in hepatocellular carcinoma cells

**Figure 1:**
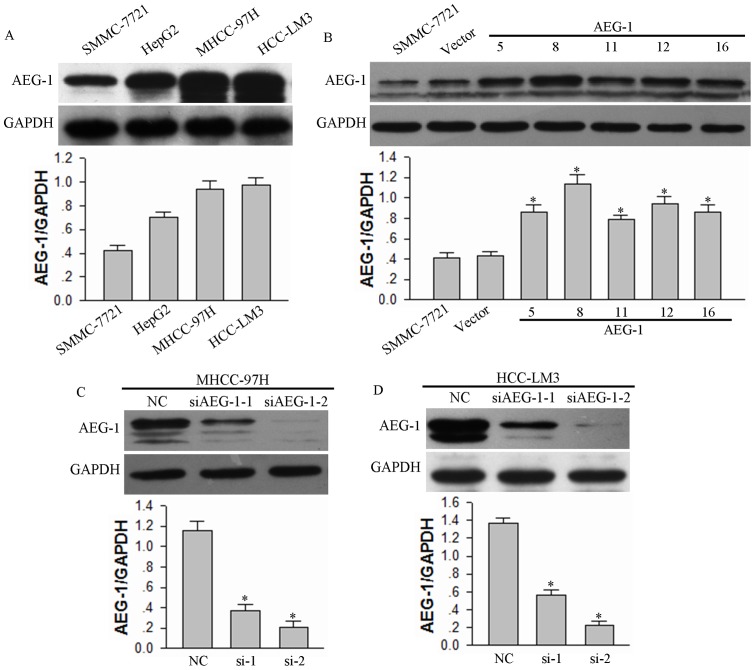
Modulation of AEG-1 expression levels in HCC cell lines. (**A**) The endogenous AEG-1 expression in four different HCC cell lines was analyzed by Western Blotting. (**B**) AEG-1 expression in SMMC-7721 cell lines transfected with pcDNA3.1(−)-AEG-1 plasmid was assessed by Western Blotting. Empty vector was transfected as a control (Vector). (**C** and **D**) AEG-1 expression in MHCC-97H and HCC-LM3 cells transfected with siRNA against AEG-1 (siAEG-1-1 and siAEG-1-2) were assessed. Scrambled siRNA was transfected as negative control (NC). GAPDH was used as a loading control for all Western blots, and the mean ±SD of the signal intensity of protein bands for AEG-1 vs. GAPDH in three independent experiments is provided for each experiment. *P<0.05 vs vector control (for panel B) or NC siRNA control (for panels **C** and **D**).

Anoikis induced by cell matrix detachment is a form of programmed cell death [41]. Conversely, anoikis resistance is a prerequisite for the survival of circulating tumor cells in tumor metastasis [42]–[43]. To characterize a role for AEG-1 in anoikis in HCC cells, we prepared HCC cells with up-regulated or down-regulated AEG-1 expression. First, we assayed the expression levels of AEG-1 protein in four liver cancer cell lines, SMMC-7721, HepG2, MHCC-97H, and HCC-LM3 (Fig. 1A). We selected SMMC-7721 cells for up-regulation of AEG-1 expression due to its relatively low expression levels, and we selected MHCC-97H and HCC-LM3 cells for down-regulate regulation of AEG-1 to provide a heterologous model cell for assessing AEG-1 function. AEG-1 protein levels in pcDNA3.1(−)-AEG-1-transfected SMMC-7721 cells were notably increased compare to empty vector-transfected cells (Fig. 1B). Furthermore, AEG-1 protein expression in MHCC-97H and HCC-LM3 cells transfected with siAEG-1-1, or siAEG-1-2 was significantly inhibited compare to cells transfected with scrambled sequence (Fig. 1C and 1D).

**Figure 2:**
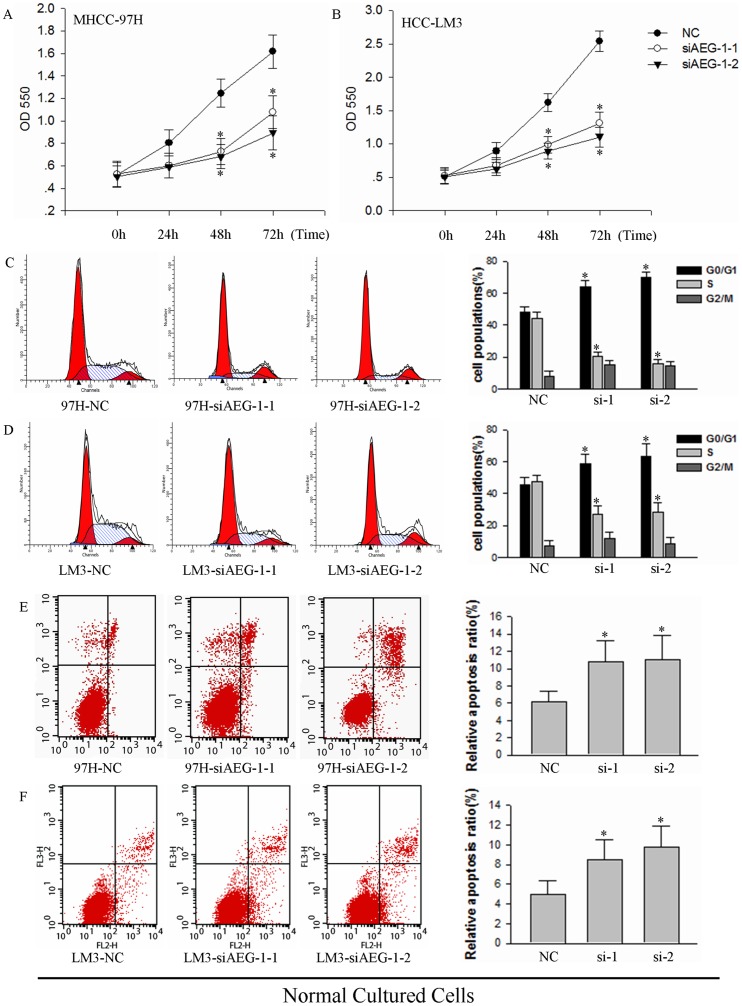
AEG-1 knockdown induces growth inhibition and cell death in MHCC-97H and HCC-LM3 cells. (**A** and **B**) Effects of AEG-1 silencing on cell proliferation were measured by MTT assay in (**A**) MHCC-97H and (**B**) HCC-LM3 cells over a time course of transfection (*P<0.05). NC, transfected with a scrambled siRNA as a negative control; siAEG-1-1 and siAEG-1-2, transfected with siRNA targeted against AEG-1. (**C** and **D**) Propidium iodide staining and cell cycle analysis was performed by flow cytometry for (**C**) MHCC-97H cells and (**D**) HCC-LM3 cells transfected with NC siAEG-1-1 or siAEG-1-2. Samples were assayed 72 h after transfection. The cytometric profile of a representative experiment which containing three (left, middle and right) cures on behalf of cell cycle (G0/G1, S, and G2/M) stage is shown (left). Area of different cures corresponding cell cycle stages in three independent experiments are quantified (right; *P<0.05). (**E** and **F**) Cell viability analysis of MHCC-97H and HCC-LM3 cells transfected with siAEG-1-1 and siAEG-1-2 for 72 h was measured. Results from a representative experiment are shown (left) and the mean±SD of three independent experiments (right; *P<0.05).

To determine effects of AEG-1 on growth, we first analyzed cell proliferation, cell cycle progression and viability of HCC-LM3 cells upon silencing of AEG-1. Compared with the negative control (NC), siAEG-1-1 and siAEG-1-2-2 both suppressed cell growth in MHCC-97H and HCC-LM3 cells as assessed by MTT assay over a timecourse of transfection. Compared with the time of 0 in MHCC-97H, reproducible 154%, 239%, 310% increase in cell growth in negative control (NC) for 24 h, 48 h and 72 h after transfection, 115%, 138%, 205% in siAEG-1-1 and 117%, 136%, 177% in siAEG-1-2-2 (Fig. 2A, *P<0.05). And compared with the time of 0 in HCC-LM3, reproducible 175%, 317%, 497% increase in cell growth in negative control (NC) for 24 h, 48 h and 72 h after transfection, 130%, 189%, 251% in siAEG-1-1 and 123%, 176%, 217% in siAEG-1-2-2. (Fig. 2B, *P<0.05). Cell cycle analysis at 72 h post-transfection showed that progression through the cell cycle was also significantly inhibited upon silencing of AEG-1: a greater percentage of cells transfected with siAEG-1-1 and siAEG-1-2 as compared to the NC were arrested in the G0/G1 phase, and the percentage of cells entering S phase were reduced remarkably (Fig. 2C and 2D). We also assessed apoptosis levels in AEG-1 knockdown cells which were cultured under normal conditions by flow cytometry. siAEG-1-1 (relative apoptosis, 10.76% and 8.45%) and siAEG-1-2 (relative apoptosis, 11.03% and 9.75%) induced increased cell death, compared to NC (relative apoptosis, 6.12% and 4.98%) in MHCC-97H and HCC-LM3 cells (Fig. 2E and 2F, *P<0.05).

**Figure 3:**
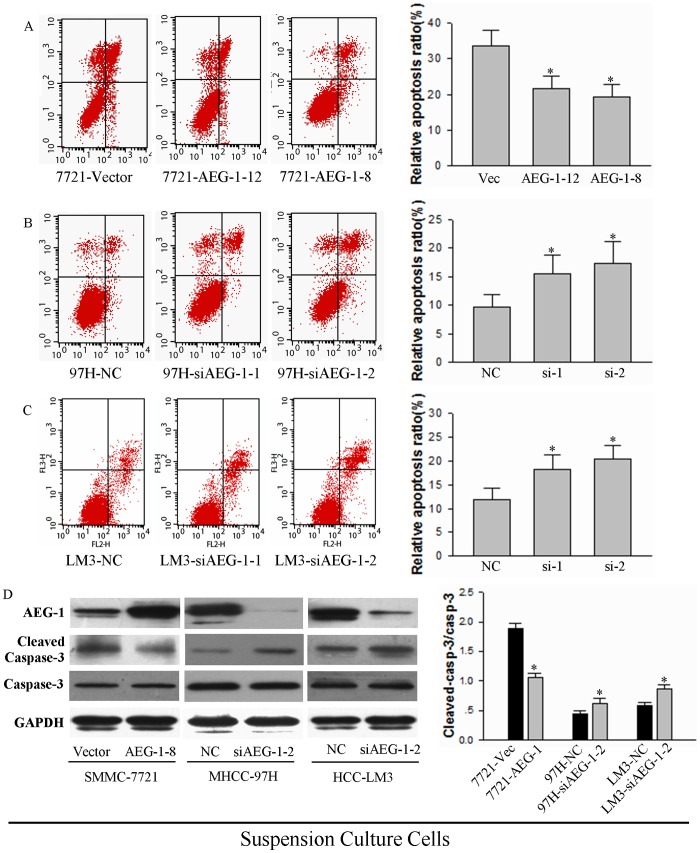
AEG-1 prevents anoikis in HCC cells. (**A**, **B** and **C**) Anoikis analysis is shown for (**A**) SMMC-7221 cells expressing either a control vector or two different clones expressing AEG-1; (**B**) MHCC-97H cells expressing either NC siRNA control or two different siRNAs against AEG-1; and (**C**) HCC-LM3 cells expressing NC or two different siRNAs against AEG-1. Cells were trypsinized and re-seeded on 2% agar precoated plates. After culture for 24 h, cells were harvested and processed by FACS analysis. Results from a representative experiment are shown (left) and results from three independent experiments (mean±SD) are quantified (right; *P<0.05). (**D**) Cleaved caspase-3 expression in AEG-1-over-expressing SMMC-7221 cells and AEG-1 knockdown HCC cells. Cells were treated as described above, and then lysates were collected and analyzed by Western blotting for one representative SMMC-7221 AEG-1 over-expressing cell line (AEG-1-8) and one representative siRNA in MHCC-97H and HCC-LM3 cells for AEG-1 knockdown (siAEG-1-2). GAPDH was as assayed as a loading control. Representative results are shown (left), and the mean +SD of the signal intensity of protein bands for cleaved (activated) caspase-3 vs uncleaved caspase-3 in three independent experiments is quantified (right; *P<0.05 vs corresponding vector or siRNA control).

We next performed experiments to examine the effect of AEG-1 expression on anoikis resistance. The AEG-1-over-expressing and AEG-1-knockdown cells were seeded on 2% agar pre-coated plates. After 24 h in suspension culture, two clones of AEG-1-over-expressing SMMC-7721 cells (AEG-1-12 relative apoptosis, 21.66% and AEG-1-8 relative apoptosis, 19.26%) underwent less cell death compared to the empty vector control cells (relative apoptosis, 33.59%). (Fig. 3A, *P<0.05). Furthermore, AEG-1 knockdown MHCC-97H (relative apoptosis, NC 9.76%, si-1 15.43% and si-2 17.28%) and HCC-LM3 (relative apoptosis, NC 11.97%, si-1 18.18% and si-2 20.39%) cells underwent more apoptosis compared to negative control (Fig. 3B and 3C, *P<0.05). These results suggest that the expression of AEG-1 correlates with the levels of anoikis resistance in two different heterologous cell models.

Anoikis, like other forms of programmed cell death, is frequently characterized by the activation of caspase-3 cleavage [44]. To verify that AEG-1 expression affects programmed cell death, we assessed caspase-3 levels in AEG-1 knockdown cells after 24 h of cell growth in suspension. A modest, but reproducible 44% decrease in levels of cleaved caspase-3 was seen in AEG-1-over-expressing SMMC-7721 cells, whereas knockdown of AEG-1 in MHCC-97H and HCC-LM3 cells reproducibly caused 32% and 25% increase in cleaved caspase-3 (Fig. 3D). These results confirm that the expression of AEG-1 is inversely correlated with anoikis in HCC cells.

### AEG-1 confers anoikis resistance via the activation of the PI3K/Akt pathway

**Figure 4:**
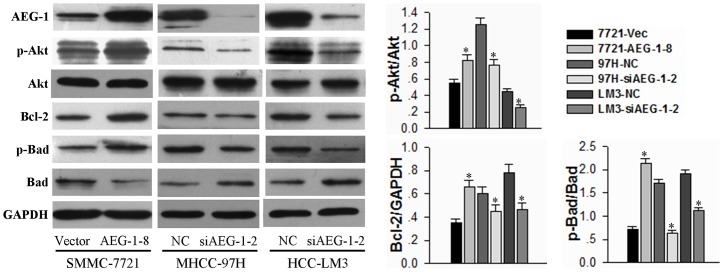
AEG-1 regulates downstream substrates of Akt in HCC cells under suspension conditions. The AEG-1-over-expressing and AEG-1-knockdown cells were grown under suspension conditions. Cell lysates were prepared 24 h after suspension culture and immunoblotted with the indicated antibodies. GAPDH is shown as a loading control. Representative results are shown (left), and the mean +SD of the signal intensity of protein bands in three independent experiments is quantified (right; *P<0.05 vs corresponding vector or siRNA control).

The PI3K/Akt signaling pathway is intimately related to anoikis resistance [11]. To determine whether AEG-1 confers anoikis resistance via the Akt pathway, we examined the phosphorylation levels of Akt (S473) after 24 h of cell growth in suspension. The AEG-1-over-expressing HCC cells (AEG-1-8) showed increased levels of phosphorylated-Akt as compared to the vector control cells. Furthermore, AEG-1 silencing in both MHCC-97H and HCC-LM3 cells resulted in significantly decreased phosphorylation of Akt compared with negative control (Fig. 4). We also examined the apoptosis-related downstream substrates of Akt to clarify the molecular mechanisms underlying AEG-1-dependent enhancement of cell anoikis resistance. The expression of the classic apoptosis regulatory protein, Bcl-2, was increased in AEG-1-over-expressing SMMC-7721 cells and decreased in AEG-1-2-silenced HCC-LM3 cells. Furthermore, the phosphorylation and inactivation of Bad, a pro-apoptotic member of the Bcl-2 family [45], had a similar pattern of correlation with AEG-1 expression (Fig. 4). These results demonstrate that modulation of AEG-1 expression affects the activation of Akt and its downstream substrates.

**Figure 5:**
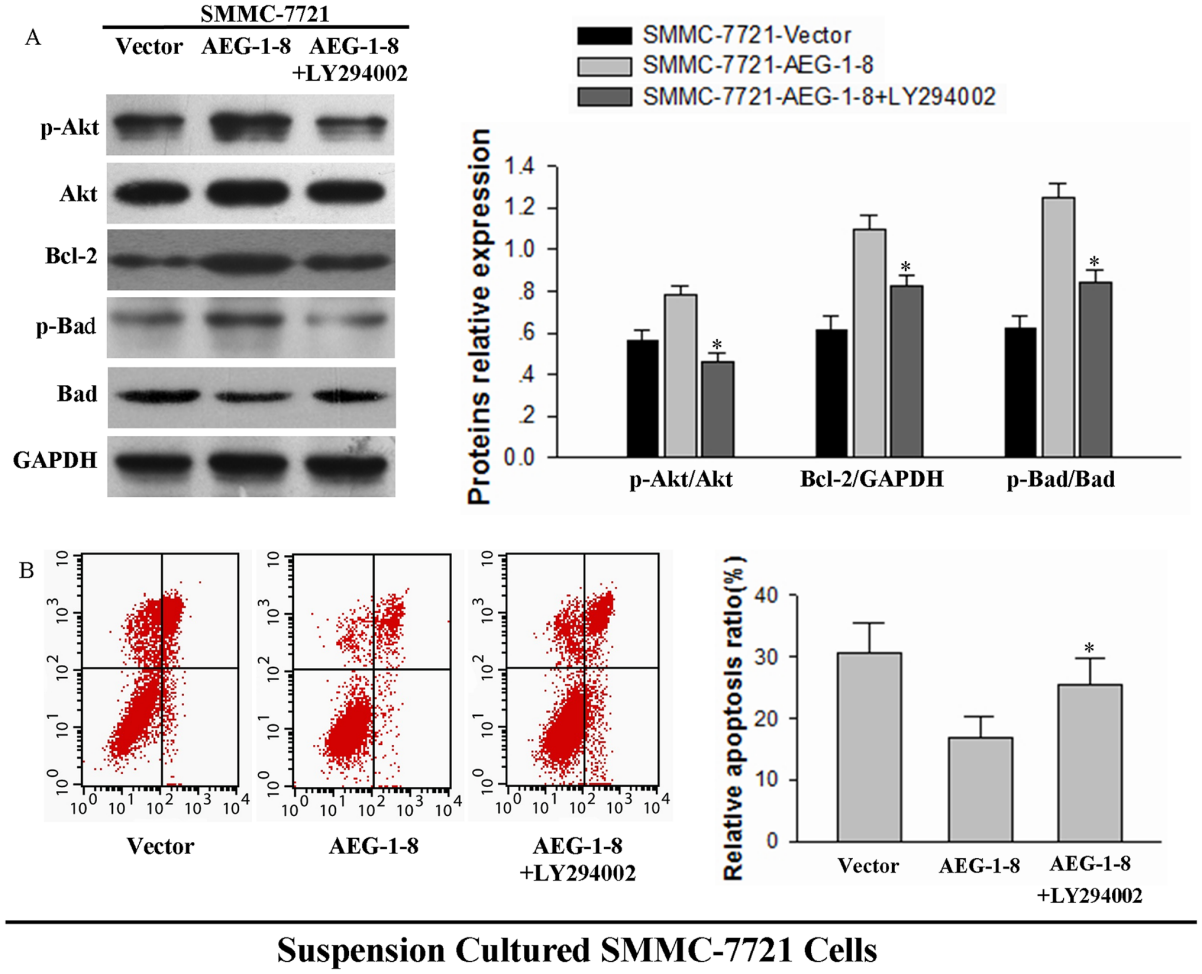
PI3K inhibitor LY294002 reverses anoikis resistance in AEG-1-over-expressing cells. (**A**) AEG-1-over-expressing cells (AEG-1-8) and control cells (Vector) were seeded onto 2% agar pre-coated plates and 50 µM LY294002 was added where indicated. Cell lysates were prepared 24 h after suspension culture and immunoblotted with the indicated antibodies. GAPDH was assessed as a loading control. Representative results are shown (left), and the mean +SD of the signal intensity of protein bands in three independent experiments is quantified (right; *P<0.05 for samples with vs without LY294002). (**B**) The above three groups of SMMC-7721 cells were cultured for 24 h in suspension. Cells were processed by FACS analysis of cell death. Results from a representative experiment (left) are shown in (**B**), and the mean±SD values of three independent experiments (right) are shown (*P<0.05).

A PI3K inhibitor, LY294002 (50 µM, Beyotime, Shanghai, China) was used to further identify the function of the PI3K/Akt signaling pathway in AEG-1-dependent anoikis resistance. LY294002 effectively reversed the increased levels of p-Akt in SMMC-7221-AEG-1 cells. Bcl-2 and phosphorylation of Bad were also inhibited by LY294002, verifying the regulation of these proteins by the p-Akt pathway (Fig. 5A). The relative apoptosis of three groups (vector, AEG-1-8 and AEG-1-8+ LY294002) were 30.57%, 16.80% and 25.42%. LY294002 reversed the attenuated levels of apoptosis in AEG-1-over-expressing SMMC-7721 cells under suspension conditions (Fig. 5B, *P<0.05). These findings support the hypothesis that the AEG-1-dependent enhancement of the PI3K/Akt pathway and consequent regulation of downstream Bcl family proteins is functionally associated with anoikis resistance.

### AEG-1 confers orientation chemotaxis to HPMECs via CXCR4/CXCL12 in HCC cells

**Figure 6:**
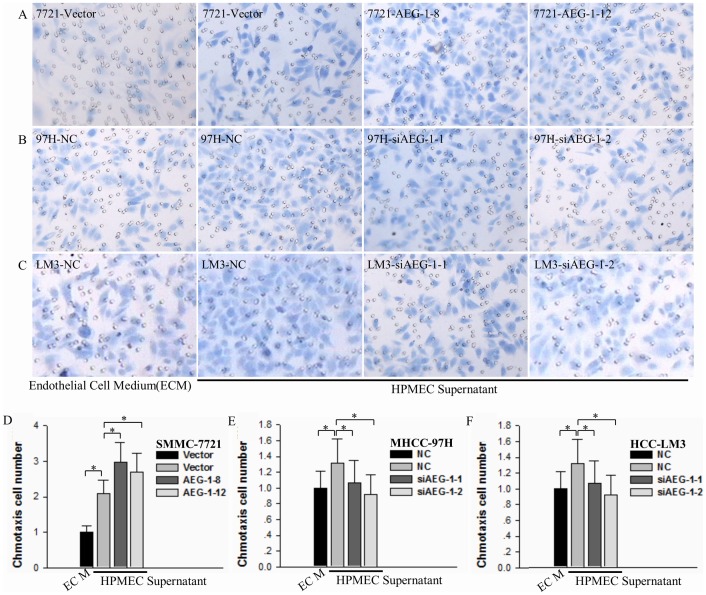
AEG-1 promotes orientation chemotaxis to HPMEC supernatant by HCC cells. (**A**) Control (Vector) and AEG-1-over-expressing SMMC-7221 cells (AEG-1-8 and AEG-1-12) were seeded onto plates that were pre-coated with 2% agar. After culture for 24 h, the suspension-cultured HCC cells were assessed for chemotaxis ability. The lower wells of a Boyden chamber were loaded with HPMEC supernatant or serum-free endothelial cell medium (ECM) as a control. Results from a representative experiment are shown (×400, HE). (**B** and **C**) MHCC-97H and HCC-LCM3 control (NC) and AEG-1 knockdown (siAEG-1-1 and siAEG-1-2) cells were assessed for chemotaxis ability as in (**A**). Results from representative experiments are shown (×400, HE). (**D**, **E** and **F**) Quantification (mean±SD) of five different fields of three independent experiments corresponding to the results in panels A-C are shown (*P<0.05).

The process of orientation chemotaxis plays a pivotal role in the initiation of metastases. To determine the effects of AEG-1 on orientation chemotaxis of anoikis-resistant HCC cells, we evaluated the ability of these cells to migrate towards chemoattractant from HPMEC supernatant in Boyden chambers. Over-expression of AEG-1 in SMMC-7721 cells significantly increased the orientation chemotaxis to HPMEC supernatant (Fig. 6A and 6D, *P<0.05). Furthermore, silencing of AEG-1 in MHCC-97H and HCC-LM3 cells significantly inhibited orientation chemotaxis (Fig. 6B, 6C, 6E and 6F, *P<0.05). These results demonstrate that AEG-1 expression modulates orientation chemotaxis in HCC cells.

**Figure 7:**
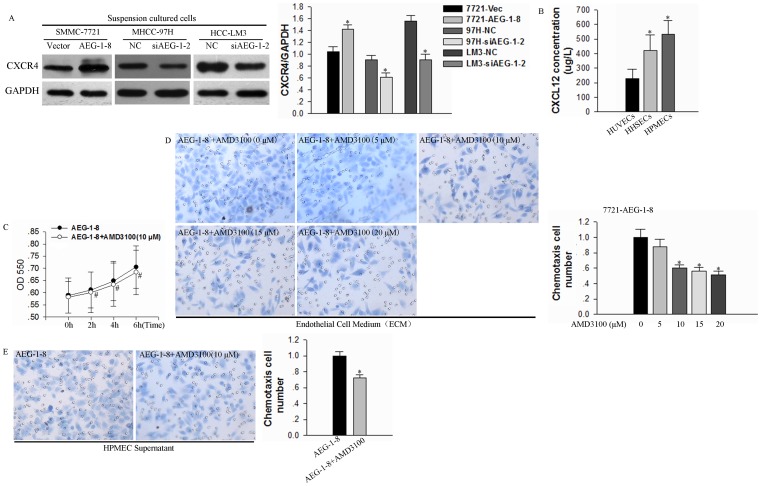
AEG-1 regulates CXCR4 expression to mediate CXCR4/CXCL12-dependent orientation chemotaxis. (**A**) To assess the role of the CXCR4/CXCL12 axis in orientation chemotaxis, HCC cells were seeded onto plates that were pre-coated with 2% again and incubated for 24h and then lysates were examined by Western blotting for CXCR4. GAPDH was tested as a loading control. Results from a representative experiment (left) and the mean±SD epressionexpression values for three independent experiments (right) are shown (*P<0.05 vs the corresponding control cell). (**B**) CXCL12 secretion in HPMECs, HHSECs and HUVECs was assessed by ELISA. Results represent the mean±SD of 3 independent experiments (*P<0.05) (**C**) Cell proliferation of AEG-1-over-expressing SMMC-7721 cells treated with or without AMD3100 (10 µM) was assessed by MTT over a timecourse of AMD3100 addition (*P>0.05). (**D**) AEG-1-over-expressing SMMC-7221 cells were cultured in suspension for 24 h and then pretreated with 0 to 20 µM or without AMD3100 from 0 to 20 (10 µM) for 2 h. Cells were assessed for chemotaxis toward HPMEC serum-free endothelial cell medium (*P<0.05) supernatant. Results from a representative experiment are shown (left) and the mean + SD of five different fields of three independent experiments are quantified (right; mean±SD; *P<0.05). (**E**) AEG-1-over-expressing SMMC-7221 cells were cultured in suspension for 24 h and then pretreated with or without AMD3100 (10 µM) for 2 h. Cells were assessed for chemotaxis toward HPMEC supernatant as above. Results from a representative experiment (left; ×400, HE), and the quantification of five different fields of three independent experiments (right) are shown (mean±SD; *P<0.05).

Recently it has been shown that tumor cells with high levels of CXCR4 expression orientation migrate to organ sites with high levels of CXCL12/SDF-1 secretion, which suggests that this molecular pair plays a key role in chemotaxis and homing of metastatic cells [46]–[47]. We, therefore, tested whether AEG-1 might function to regulate the CXCR4/CXCL12 axis. Western analysis of suspension cultured cells showed that CXCR4 was up-regulated in SMMC-7721 cells with AEG-1 over-expressing and down-regulated in MHCC-97H and HCC-LM3 cells with AEG-1 knockdown (Fig. 7A). ELISA analysis of 48 h-cultured supernatants verified the secretion of CXCL12 for these microvascular endothelial cells (HHSECs, HUVECs and HPMECs), and also showed that HPMECs supernatant displayed the highest concentration of CXCL12 (Fig. 7B).

To verify the function of CXCR4 in mediating AEG-1-dependent chemotaxis, we examined the effects of AMD3100, a specific CXCR4 antagonist [48]. 10 µM AMD3100 administration had no growth inhibitory effect on growth-inhibitory. Compared with the time of 0, reproducible 104%, 110%, 120% increase in cell growth in AEG-1-8 for 2 h, 4 h and 6 h cell culture, and 105%, 111%, 119% in AEG-1-8 plus with 10 µM AMD3100 (Fig. 7C, *P>0.05). The addition of AMD3100 partly reduced orientation chemotaxis to HPMEC supernatant in SMMC-7721-AEG-1 cells (Fig. 7D and 7E, *P<0.05). These results support the function of CXCR4/CXCL12 in mediating AEG-1-dependent orientation chemotaxis.

## Discussion

The present studies uncover a uncharacterized mechanism by which elevated endogenous AEG-1 contributes to anoikis resistance in HCC cells though the activation of the PI3K/Akt signaling pathway and contributes to chemotaxis in HCC cells via the CXCR4/CXCL12 axis. Anoikis resistance and chemotaxis are important features of metastatic tumors, and our research verifies that AEG-1 plays an important role in the metastasis of HCC cells. To provide increased support for our results, we up-regulated AEG-1 in a HCC cell line with low endogenous levels and down-regulated AEG-1 expression in two HCC cell lines with high endogenous levels for verification in heterologous model systems of AEG-1 function.

Normal epithelial cells require cell-extracellular matrix contacts for survival and proliferation, and disruption of these contacts induces anoikis, a type of programmed cell death [11]. Anoikis resistance, which allows circulating tumor cell survival in the blood stream, is a prerequisite for the development of tumor metastasis [49]. Previous studies have validated that AEG-1 is dysregulated in HCC and promotes the metastasis of HCC cells [16]. Over-expression of AEG-1 augments the anchorage-independent growth of HeLa cells and human glioma cell lines and increases their migration and invasion properties [50]–[51]. These data suggest that AEG-1 may be involved in anoikis resistance in tumor cells. Our research revealed a significant anoikis resistance in AEG-1-over-expressing cells under suspension culture. A number of published observations have demonstrated the important role of the PI3K/Akt pathway in the activation in anoikis resistance [52]–[54]. Three primary integrin signaling molecules, FAK (focal adhesion kinase), Shc and ILK (integrin-linked kinase), have been linked to cell survival and may activate the PI3K/Akt pathway [55]–[57]. A previous study showed that the PI3K/Akt pathway is not only activated by AEG-1 but also plays a key role in regulating AEG-1 expression [58]. AEG-1 also mediates the survival of primary human fetal astrocytes under serum starvation via activation of the PI3K/Akt pathway [20]. We have shown that AEG-1 significantly activates the phosphorylation of Akt in SMMC-7721 cells under suspension culture and the PI3K inhibitor LY294002 reduces the AEG-1-mediated anoikis resistance. We also found that the expressions of the apoptosis-related molecules Bcl-2 and Bad, which are downstream mediators of the Akt pathway, were affected by AEG-1 expression. The regulation of survival signaling in anoikis is also likely to be affected by cytoskeletal changes apparent in transformed cells [59]. Numerous studies have validated that anoikis can be inhibited when cell-cell adhesion is preserved in various epithelial carcinoma cell lines [60]–[61]. The mouse/rat orthologue of AEG-1 was found to encode the lysine-rich CEACAM-1 co-isolated protein (Lyric) that co-localizes with the tight junction protein ZO-1 in polarized rat prostate epithelial cells [62]. AEG-1 also has been suggested to regulate the expressions of β-catenin [16], ICAM2, ICAM3, selectin E and selectin P [63], all of which are associated with the cell-extracellular matrix or cell-cell adhesion. Further studies will be necessary to identify additional mechanism by which AEG-1 may regulate anoikis.

While anoikis resistance is a prerequisite for survival of circulating tumor cells, metastasis also involves the acquirement by anoikis-resistant circulating tumor cells of the ability to chemotax to target organs [64]. Our previous study verified that the expression of AEG-1 in HCC cell lines is positively correlated with orientation chemotaxis to HPMECs [22]. Herewithin, we have provided the first demonstration that modulation of AEG-1 expression, by either over-expression or knockdown, directly affects orientation chemotaxis in HCC cells. These results suggest that AEG-1 plays an important role in the regulation of HCC orientation chemotaxis.

Stromal cell-derived factor-1 (SDF-1 or CXCL12) and its unique receptor CXCR4 have prominent roles in chemotaxis and metastasis of a diverse number of cancers [46]–[47]. CXCL12 is a small, secreted peptide that is involved in the initiation and development of many tumors through its role in organ-specific metastasis [65]. CXCR4 is a seven-transmembrane, G protein-coupled receptors that is found to be expressed in many human cancer cells [33]–[35]. We assessed the involvement of this receptor/ligand pair in chemotaxis. Our results showed that HPMEC cells, which promote the orientation chemotaxis of SMMC-7721 cells, secrete relatively high levels of CXCL12 as compared with HHSECs and HUVECs. We demonstrated that CXCR4 expression is affected by the modulation of AEG-1 expression, providing the first demonstration that CXCR4 is downstream of AEG-1. Furthermore, the CXCR4 antagonist AMD3100 partly decreased chemotaxis to HPMEC supernatant, further supporting the functional role of CXCR4. These results are consistent with previous evidence demonstrating that selective inhibition of CXCR4 suppresses CXCL12-induced migration of cancer cells, invasion, neo-angiogenesis and metastases [48]. Therefore, our data suggests that AEG-1 promotes the orientation chemotaxis of suspension-cultured cells to HPMECs, at least in part, through the CXCR4/CXCL12 axis. Given that CXCR4 and CXCL12 have been shown to have an additional role in anoikis in breast and colorectal cancers [36]–[37], future studies addressing the possible possibility that the upregulation of CXCR4 by AEG-1 may also function to regulate anoikis are of interest.

In conclusion, these results directly demonstrate the effects of AEG-1 on anoikis resistance and orientation chemotaxis in HCC cells and also characterize the molecular pathways by which these processes are activated to enhance tumor cells metastasis. A better understanding of the factors that regulate the complex metastatic process may lead to the discovery of new targets for anti-metastasis therapy.
